# The Use of Organs from Executed Prisoners in China

**Published:** 2012-04-30

**Authors:** Rosalba Romano, Ornella Piazza

**Affiliations:** Department of Medicine, Salerno University

**Keywords:** NHBD, transplantation ethics

During a conference held in Hangzhou on the 15^th^ of March 2012, Huang Jiefu, China’s Deputy Minister of Health, announced that China will ban the transplantations of human organs from executed prisoners in 3 to 5 years [1].

It represents the first step to regulate organ transplantation in a Country where 65% of transplants are grafts from non-heart-beating donors, over 90% of whom are executed prisoners. Living donors provide to the other 35% of transplantations. Donations from heart-beating donors, whose death is declared by neurological criteria (Harvard criteria 1968), are lacking. In 2003 China’s Ministry of Health investigated the possibility to diagnose brain death, but the poor understanding of the concept by public and even by physicians have made the legislation about brain death abandoned. Since 2007 a pilot organ donation programme has run in 10 provinces, including Shangai [2]. This programme is explicated in the Regulation on Human Organ Transplantation, which aim is to reduce the amount of grafts from executed prisoners and to encourage donations from citizens. The lack of volunteer donations is related to the Confucians’ rule to save the integrity of the body after death, but a survey among students demonstrates that things are changing. The Regulation on Human Organ Transplantation has not banned the practise of harvesting organs from executed prisoners, but has condemned illegal trade and transplantation tourism [3]. The reduction of transplants from executed prisoners has increased the amount of donations from living donors. The Regulation approves only donations from relatives to avoid a legal trade of grafts.

Lately the indignation about Chinese shameful trade of organs has been growing. In 2001 a protest was presented to the committee on international relations of United States House of Representatives in Washington to enlighten the use of grafts from executed prisoners. Harvested organs are transplanted into recipients from all over the world. Patients come from the United States, Southeast Asia, Europe and Australia and pay $17 000 – 40000 each. In China prisoners could be executed for many crimes such as murder but also rape, robbery, drug dealing, and black market activities [4].

Amnesty International maintains that in 2008 alone 1,718 prisoners were put to death [2].

In 2009 the documentary “H.O.T.- Human Organ Traffic” reports interviews with people involved in organ traffic all over the world. It shows what happens in various Countries and focuses attention on organ retrieval from Chinese prisoners. The aim of the director is informing and making aware the scientific community and lay people [5].

Organs are harvested from prisoners immediately after death by surgeons waiting at the site of executions. The cause of death is described as a “severe brain injury” which should be referred to a gun-shot to the head. Witness reported continued movements and spontaneous respirations in some prisoner-donors. The China Liver Transplant Registry reported 18375 recipients from January 1993 until July 2010 [6].

Allam et al showed that the amount of patients who received a transplant in China is increased because of the growing need of organs. The Authors compared the outcome of patients transplanted in China and in Egypt. Patients were included in waiting list for liver transplantation in their own countries or, on the contrary, their request had been denied for unsuitable medical condition as co-morbidities, advanced age or the presence of an advanced hepatocellular carcinoma. Patients were transplanted in China because transplantation was cheaper and easier, the waiting time was shorter and the transplantation indications were wider. The waiting time in China ranged from 5 to 20 days. Donors were from 20 to 35 years old [7].

Mr. Huang, who is a surgeon and has been the director of The Hepatic Surgery at Sun Yat-Sen College of Medical Science, No. 1 Hospital (as reported in www.chinavitae.com), has legitimated the decision of reducing transplantation from executed prisoners referring not to ethical but medical issue. He maintains that the rates of fungal and bacterial infection in organs taken from executed prisoners are more frequent and that the long-term survival rates of recipients are worse than the rates of recipients in other Countries [1].

China is the only country to use systematically organs from executed prisoners in transplantation procedures. This is our opportunity to express condemnation for the use of prisoners’ organs and the violation of human rights and medical ethics.
